# Proton pump inhibitors can reverse the YAP mediated paclitaxel resistance in epithelial ovarian cancer

**DOI:** 10.1186/s12860-019-0227-y

**Published:** 2019-11-12

**Authors:** Jing He, Xiao-Yan Shi, Zhi-min Li, Xiao-hua Pan, Ze-Lian Li, Ying Chen, Shi-Jie Yan, Lan Xiao

**Affiliations:** 1 Department of Obstetrics & Gynecology, First Affiliated Hospital, An Hui Medical University, Hefei, 230020 Anhui People’s Republic of China; 2 Anhui Province Key Laboratory of Reproductive Health and Genetics, Hefei, 230020 Anhui People’s Republic of China; 3 Department of Obstetrics and Gynecoloy, An Qing Municipal Hospital, An Qing, 246003 AnHui People’s Republic of China; 4 Central Laboratory, The Central Hospital of Wuhan, Tongji Medical College, Huazhong University of Science and Technology, Wuhan, 430014 Hubei People’s Republic of China; 5 Department of Gynecology, Guangdong Women and Children Hospital, Guangzhou, 510010 Guangdong People’s Republic of China

**Keywords:** Vacuolar H + -ATPase, YAP, Proton pump inhibitors (PPI), Paclitaxel resistance, Epithelial ovarian carcinoma (EOC)

## Abstract

**Background:**

Several reports indicated that the expression of Yes-associated protein (YAP) was associated with multi-drug resistance. Acidic microenvironment increased by the overexpression of vacuolar-ATPase (V-ATPase) was also observed in tumor growth and drug resistance. We hypothesize that proton pump inhibitors (PPIs), currently used in the anti-acid treatment of peptic disease, could inhibit the acidification of the tumor microenvironment and increase the sensitivity of tumor cells to cytotoxic agents. Thus, our objective is to explore the reversal of drug resistance by the inhibition of YAP through specific PPIs in the epithelial ovarian carcinoma (EOC) cells. .

**Results:**

We found that V-ATPase D1 was a positive regulator of YAP. Sub-lethal doses of the proton pump inhibitor esomeprazole (EMSO) in combination with paclitaxel (PTX) increased the PTX sensitivity in PTX-resistant EOC cells, as compared to PTX single treatments by inhibiting YAP and reserving pH gradient created by the V-ATPase D1. Moreover, sub-lethal doses of EMSO combined with PTX decreased autophagy and improved caspases independent apoptosis of PTX-resistant EOC cells.

**Conclusions:**

These results suggested that sub-lethal doses of esomeprazole reverse YAP-mediated PTX resistance through the inhibiting of both YAP expression and acidic tumor microenvironment created by the V-ATPase D1. Therefore, we think the use of PPIs represents a promising strategy to improve the effectiveness of anti-EOC.

## Background

In tumor cells, YAP (Yes-associated protein) is closely associated with tumorigenesis as one of the major transcription activation factors, plays an important role in regulating cell proliferation and organ development [1]. YAP is overexpressed, and is connected with multi-drug resistance [2, 3]. Our previous study [4] demonstrated that YAP was highly expressed in cisplatin-resistant EOC cancer cells. As a new anti-tubulin drug [5], paclitaxel (PTX) can raise the binding ability of 14-3-3 protein with YAP while remaining the stability of tublins and maintaining YAP in a phosphorylation status [6]. However, the inhibition of PTX by YAP can be reversed by the up-regulation of YAP [7].

Cancer cells survival and proliferation in competition with somatic cells and according to the physical and biological properties of their microenvironment. The constant high level of glycolytic activity in tumor cells leads to an increased production of lactic acid and a decreased pH of the extracellular microenvironment. Thus, in tumor cells, V-ATPases maintains an appropriate relatively neutral intracellular pH and an acidic extracellular pH [8]. The acidic microenvironment increased by overexpression of V-ATPases is observed in tumor growth, metastasis and chemoresistance [9]. In addition, several studies have demonstrated that PPIs (proton pump inhibitors), which directly inhibit V-ATPase at the cellular level, has reverted chemoresistance of drug-resistant tumors [10–12]. YAP/TAZ activation and a shift toward a glycolytic metabolism are commonly observed during tumor progression [13]. YAP is regulated by diverse mechanisms including microenvironmental factors [14]. V-ATPase D1 also known as ATP6V0D1, is the D subunit of the V0 domain. Expressed ubiquitously, V-ATPase D1 acts in concert with other V0 subunits to catalytically acidify a variety of intracellular compartments. V-ATPase D2 has been implicated as a regulator of urine acidification, osteoclast fusion and bone formation. Furthermore, V-ATPase D2 has been identified as a dendritic cell marker. Therefore, we wondered whether the inhibition of V-ATPase D1 through PPI can reserve the PTX resistance caused by the over-expression of YAP, and this may lead to the clinical use of PTX.

In the current study, we find a novel role of V-ATPase D1 in correlation with YAP. PTX resistance of the EOC cancer cells can be reversed by EMSO co-treatment and the YAP inhibition. Furthermore, we uncovered a downloaded of a novel V-ATPase D1/YAP signaling pathway, which may be targeted in EOC treatment. Thus, we have a clinical proof of concept that PPI may well be included in new anti-cancer strategies.

## Results

### YAP is highly expressed in EOC and is directly regulated by V-ATPase D1 expression

**Fig. 1:**
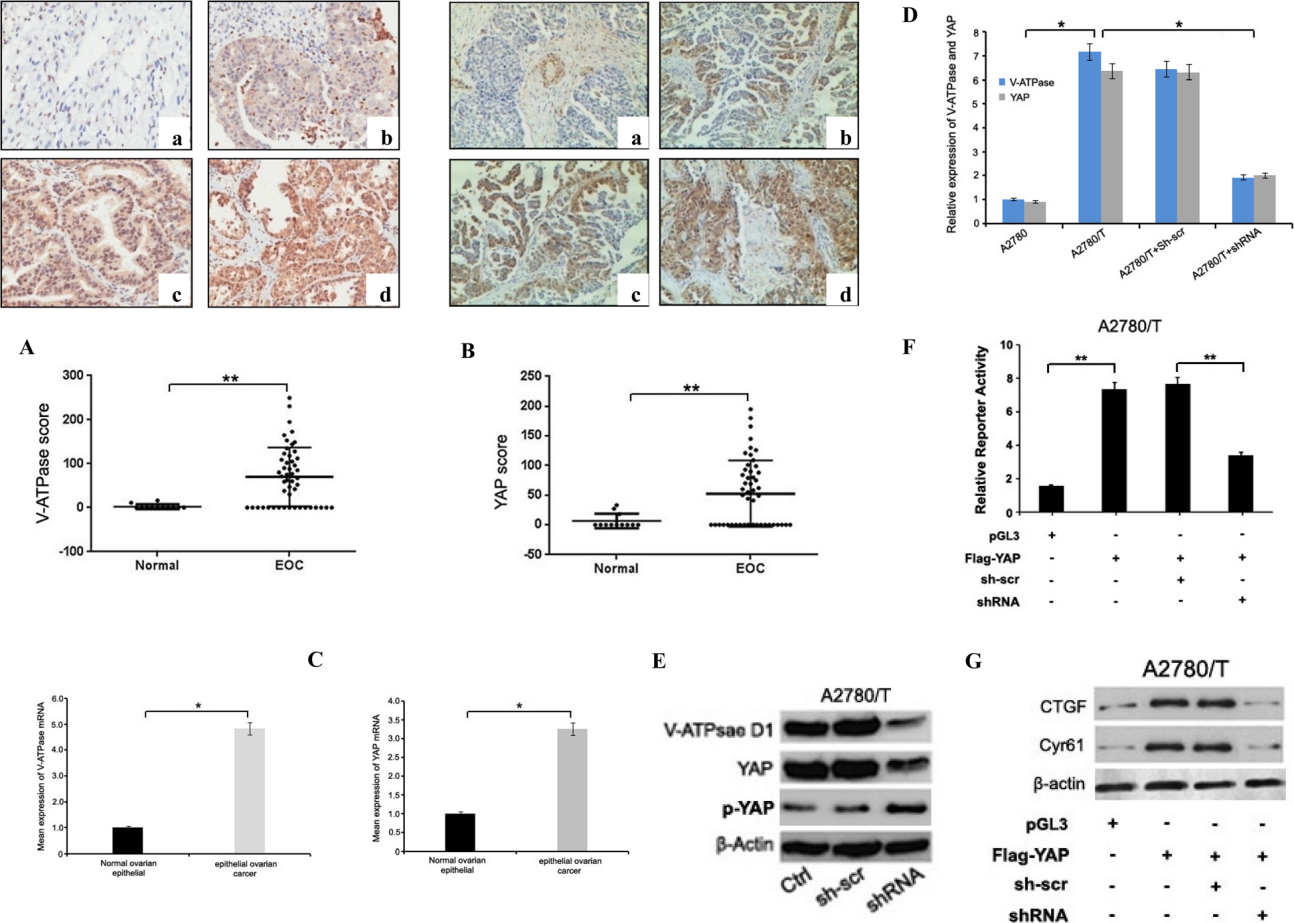
YAP is highly expressed in EOC and is directly regulated by V-ATPase D1 expression. Immunohistochemical observation of V-ATPase D1 and YAP protein expression in normal ovarian epithelium and EOC tissues, **a** V-ATPase D1 and **b** YAP (with extremely weak staining (0), a weak staining (+ 1), a moderate staining (+ 2), a strong staining (+ 3). All photographs were taken at original 200 × magnification. The scores of (**a**) V-ATPase D1 and (**b**) YAP by beeswarm plot was shown in the bottom (**P < 0.01). **c** The mRNA level of V-ATPase D1 and YAP in normal ovarian epithelium and EOC tissues were determined using a real-time PCR kit. Data represent the mean and standard deviation (mean ± SD) are from three independent experiments (*P < 0.05). **d** V-ATPase D1 and YAP expression assessed by Q-PCR analysis in EOC cell lines. Data (mean ± SD) are from three independent experiments (*P < 0.05). **e** V-ATPase D1, YAP and p-YAP protein levels in A2780/T cells were performed by western blot. **f** Knocking down the expression of V-ATPase D1 in A2780/T cells impaired the transcriptional activity of YAP in the luciferase assay. **g** Knocking down the expression of V-ATPase D1 in A2780/T cells impaired the binding of YAP to the promoter region of CTGF and Cyr61

We found that both V-ATPase D1 and YAP were over-expressed in EOC tissues. V-ATPase D1 was predominantly observed in the cytoplasm of the cells (Fig. 1a). V-ATPase D1 expression was observed only in two normal ovarian epithelium but was expressed in 33 of 50 EOC tissues (66%). Immunohistochemical analysis of YAP positive expression was detected in both nuclear and cytoplasm cell compartments. The positive expression rates of YAP in EOC tissues were 56% (28/50) (Fig. 1b). High nuclear YAP levels were observed in ovarian tumor samples than nuclear V-ATPase D1 levels. The normal ovarian epithelium just showed weak staining for YAP in the cytoplasm. The expression of V-ATPase D1 correlated with YAP expression in the EOC tissues (*r* = 0.467, *P* < 0.01). The expression levels of V-ATPase D1 and YAP mRNA were higher in EOC tissues, compared with normal ovarian epithelium tissues (*p* < 0.05, Fig. 1c). Since V-ATPase D1 expression was increased in PTX-resistant EOC cells, we next examined the effect of V-ATPase D1 silencing on YAP levels in PTX-resistant A2780/T cells. We used a short hairpin RNA against V-ATPase D1(shRNA) or a non targeting scrambled short hairpin RNA (sh-scr). Representative data from A2780/T cells is shown in Fig. 1d. The V-ATPase D1 shRNA transfected cells exhibited 3.34-fold and 3.16-fold inhibition at V-ATPase D1 and YAP mRNA levels, compared with scrambled control, respectively (Fig. 1d). The V-ATPase D1 silencing was further confirmed at protein level, a 3.60-fold and 2.25-fold reduction in V-ATPase D1 and YAP, a 1.95-fold promotion of p-YAP protein expression were observed by western blotting, respectively (Fig. 1e). As shown in Fig. 1f, downregulation of V-ATPase D1 impaired the transcriptional activity of YAP in the luciferase assay in A2780/T cells. Knocking down the expression of V-ATPase D1 in A2780/T cells impaired the expression of target proteins downstream of YAP (Fig. 1g). These results suggested that V-ATPase D1 activated the transcriptional activity of YAP in PTX-resistant A2780/T cells.

In addition, a comparison conducted between stages of ovarian cancer showed that the levels of expression of YAP and V-ATPase D1 were significantly higher in stage III-IV, compared to stage I-II. The high levels of YAP and V-ATPase D1 proteins were positively associated with lymphatic invasion, tumor grade, and tumor size in EOC patients (Tables 1 and 2).

**Table 1:** Association of V-ATPase D1 expression with clinicopathological factors in epithelial ovarian carcinoma patients

Features		n	V-ATPase D1		χ2	P
			Positive	Negative		
Mean age	> 55	31	21	10	0.110	0.740
	≤55	19	12	7		
FIGO stage	I-II	12	3	9	11.828	0.001
	III-IV	38	30	8		
Lymphatic invasion	Positive	34	28	6	12.662	0.000
	Negative	16	5	11		
Pathological type	Mucinous	14	8	6	2.247	0.325
	Serous	25	19	6		
	Others	11	6	5		
Tumor grade	G1	10	2	8	14.925	0.001
	G2	24	16	8		
	G3	16	15	1		
Tumor size	<10	34	18	16	8.074	0.004
	≥10	16	15	1

**Table 2:** Association of YAP expression with clinicopathological factors in epithelial ovarian carcinoma patients

Features		n	YAP		χ2	P
			Positive	Negative		
Mean age	> 55	31	17	14	0.045	0.833
	≤55	19	11	8		
FIGO stage	I-II	12	1	11	14.560	0.001
	III-IV	38	27	11		
Lymphatic invasion	Positive	34	26	16	11.325	0.001
	Negative	16	2	14		
Pathological type	Mucinous	16	9	7	3.852	0.146
	Serous	24	16	8		
	Others	10	3	7		
Tumor grade	G1	10	2	8	12.054	0.002
	G2	24	12	12		
	G3	16	14	2		
Tumor size	<10	36	15	21	10.720	0.001
	≥10	14	13	1		

### YAP-TEAD1 confers PTX-resistance to EOC cells

**Fig. 2:**
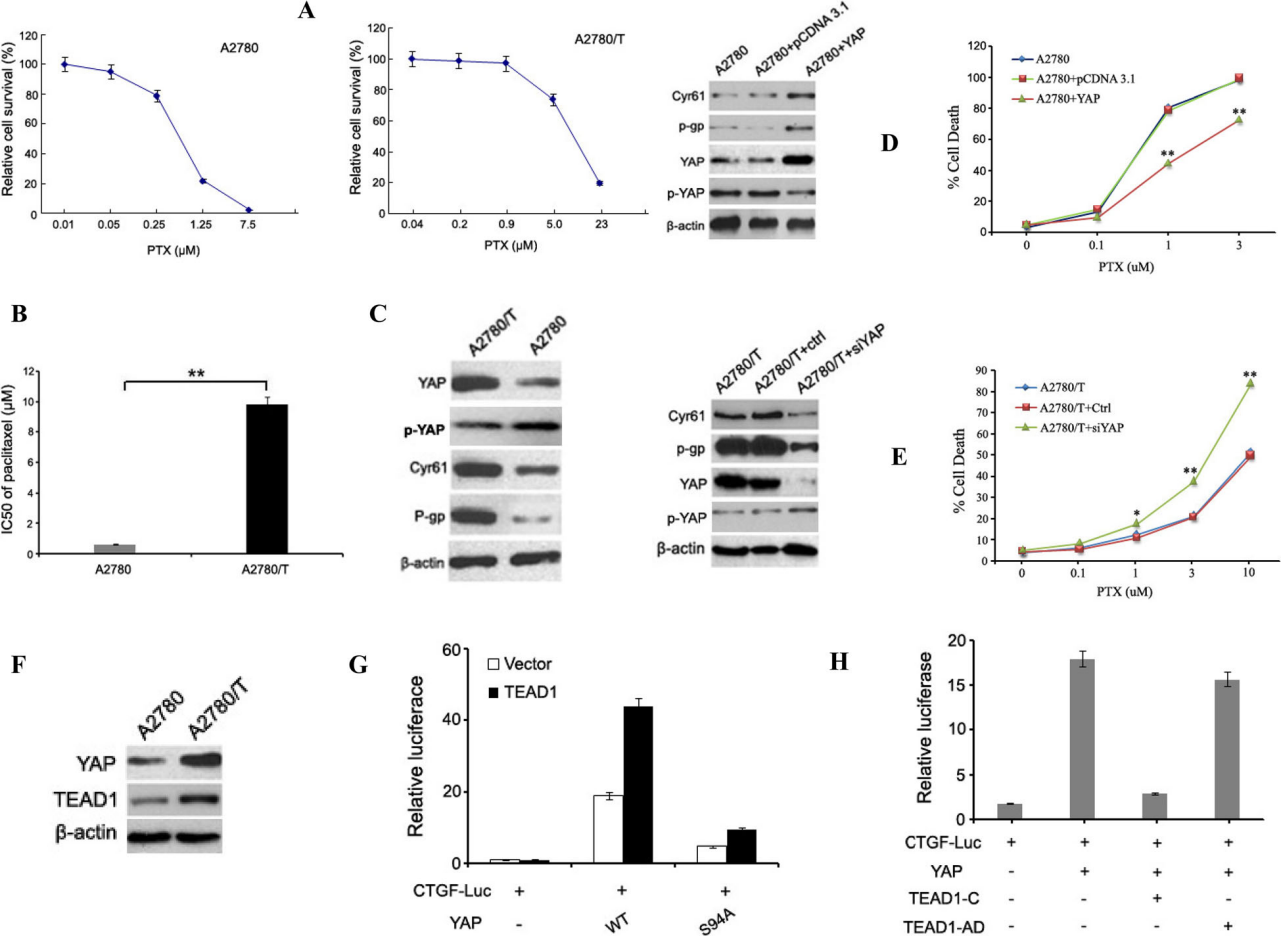
YAP-TEAD1 confers PTX-resistance to EOC cells. Cells were treated with different concentrations (0-23 μmol/L) of PTX for 48 h. **a** Dose-response curve of PTX-sensitive and PTX-resistant EOC cells. **b** The 50% maximal inhibitory concentration (IC_50_) values of PTX in the PTX-sensitive parent cell line A2780 and its resistant clone A2780/T were calculated. Each value represents the mean ± SD from three independent experiments (**P < 0.01). **c** YAP, p-YAP, Cyr61 and P-gp protein levels were determined by western blot. **d-e** Western blotting analyses of YAP, p-YAP, Cyr61 and P-gp protein expression in YAP-low/ PTX-sensitive A2780 cells and in YAP-high/PTX-resistant A2780/T cells by plasmids expressing His-tagged YAP and YAP siRNA stable transfection, respectively. CCK-8 assay indicated that over-expression of YAP in A2780 cells significantly reduced its drug-sensitivity to PTX while knocked down of YAP in A2780/T cells sensitized their response to PTX treatments. Each value represents the mean ± SD from three independent experiments (**P < 0.01). **f** Western blotting analyses of TEAD1 protein expression in A2780 and A2780/T cells. **g** Activation of CTGF reporter by YAP and TEAD1. A luciferase reporter driven by CTGF promoter was co-transfected with YAP wild type or S94A mutant as indicated with or without TEAD1 co-transfection. Luciferase activity was measured and normalized to co-transfected β-galactosidase. **h** Dominant-negative TEAD1 blocks the YAP stimulation of the CTGF reporter. The indicated plasmids were co-transfected, and luciferase activity was determined as in G

The stability of PTX-resistant in A2780/T cells were compared to PTX-sensitive A2780 cells was confirmed by dose-response curves (Fig. 2a). CCK-8 assay showed that A2780/T achieved a high degree of resistance to PTX. The 48-h IC50 with PTX of the A2780 and A2780/T cells was 0.5 ± 0.12 μmol/L and 9.3 ± 0.5 μmol/L, respectively (Fig. 2b). As shown in Fig. 2c, the expression of the corresponding proteins, P-gp, Cyr61, YAP were overexpressed and lower p-YAP expressions in the A2780/T cells, compared with the A2780 cells. To further confirm the relationship between YAP and PTX resistance, we firstly over-expressed in YAP-low/PTX-sensitive A2780 cells (Fig. 2d), using plasmids expressing His-tagged YAP. Over-expression of YAP in A2780 cells induced a promotion of Cyr61, p-gp, YAP, and a suppression of p-YAP protein expression. Obviously, over-expression of YAP and lower p-YAP expression in A2780 cells significantly reduced its drug-sensitivity to PTX (Fig. 2d). Secondly, we knocked down YAP gene in YAP-high/PTX-resistant A2780/T cells by using siRNAs. YAP silencing in A2780/T cells significantly suppressed Cyr61, p-gp, YAP and upregulated p-YAP protein expression. Correspondingly, YAP silencing in A2780/T cells sensitized theirs response to PTX treatments (Fig. 2e). Taken together, these results strongly confirmed that YAP is a biomarker for the resistant of EOC cells to PTX.

TEAD family members are YAP downstream co-activators. Multiple genes strongly correlated with tumorigenesis were regulated by TEAD family, including CTGF and Cyr61. Our results showed that Cyr61 and CTGF were highly induced by YAP expression in A2780/T cells, compared to A2780 cells (Fig. 1g). In ovarian cancer initiated cells, TEAD1 and TEAD4 were found to be expressed at significantly higher levels than in differentiated ovarian cancer cells. In this study, we focused on YAP downstream co-activators TEAD1. As shown in Fig. 2f, the expression of TEAD1 was overexpressed in the A2780/T cells, compared with the A2780 cells. Next, we cloned the CTGF promoter into a basic luciferase reporter and found that it was potently activated by YAP but not by YAP-S94A, and the activation was further enhanced by TEAD1 co-expression (Fig. 2g). Expression of the dominant-negative TEAD1-C, but not the TEAD1-C-AD (in which the C-terminal YAP-binding domain was replaced by the YAP transactivation domain), blocked the activation of CTGF reporter by YAP (Fig. 2h). These results indicate that YAP activates the CTGF promoter through TEAD1.

### Effects of acidic pHe on V-ATPase D1 change

**Fig. 3:**
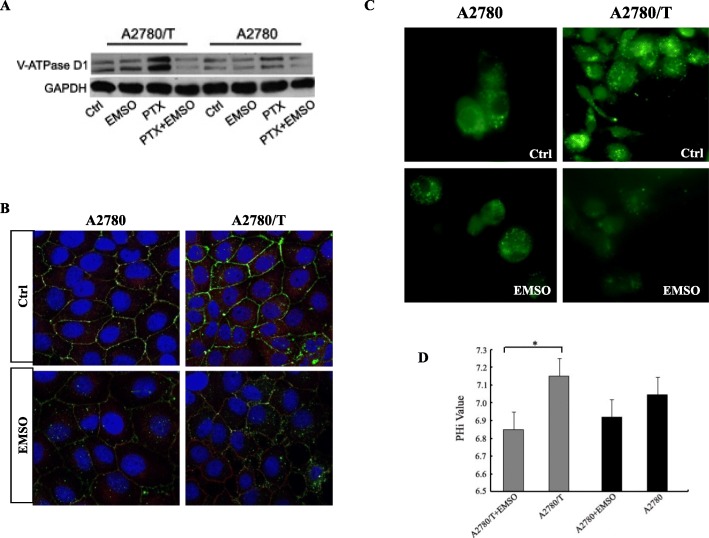
Effects of acidic pHe on V-ATPase D1 change. **a** Total protein from PTX- resistant EOC cells and respective sensitive phenotypes were immuno-blotted with anti-V-ATPase D1. **b** Confocal microscopy showing V-ATPase D1 (green dots) and plasma membrane (red/orange staining) in A2780 and A2780/T cells. DAPI in blue color represents nuclear staining. Merged images (yellow regions). Original magnification× 600. Representative images from three independent experiments are shown. **c** Cytosolic pH was estimated using pH sensitive dye BCECF-AM. Significantly decreased intracellular pH was observed in EMSO co-treated with PTX in A2780/T cells using BCECF-AM and fluorescence microscopy (488/535 nm, Magnification× 600). **d** The corresponding intracellular pH was obtained from the pH calibration curve. Values are means ± S.E.M of two independent experiments performed in triplicate (*P < 0.05)

PTX combination with sub-lethal doses of EMSO (130 and 150 μM) for 48 h induced a significantly expression decrease in V-ATPase D1 protein levels in A2780/T cells and A2780 cells (Fig. 3a). Confocal microscopy analysis confirmed the co-localization of V-ATPase D1 with the plasma membrane marker, pan-cadherin, suggesting that the isoform is expressed on the plasma membrane of both PTX-sensitive and PTX-resistant tumor cells, with elevated expression in PTX-resistant cells. Sub-lethal dose of EMSO for 48 h did induce a reduction of V-ATPase D1 protein expression in both cell membrane and cytosolic compartments of A2780/T cells, as compare to control cells. V-ATPase D1 levels were slightly reduced in A2780 cells with sub-lethal dose of EMSO (Fig. 3b). The change in pH in two EOC cells was confirmed by EMSO treatment. Alterations in intracellular pH in two EOC cells were verified using the BCECF-AM pH indicator. Fluorescence was significantly decreased in PTX-resistant A2780/T cells, indicating that intracellular pH was acidified by V-ATPase D1 inhibition. In contrast, intracellular pH showed no significant change in PTX-sensitive A2780 cells (Fig. 3c). Quantitative analysis showed that the intracellular pH decreased in A2780/T cells, but statistical significance was not achieved in A2780 cells (Fig. 3d). In summary, in both A2780/T and A2780 cells, V-ATPase D1 was co-localized in the cell plasma and membranes. Notably, we found higher plasma and membrane V-ATPase D1 expression in PTX-resistant A2780/T cells than PTX-sensitive A2780 cells. The A2780/T cells showed higher acidification activity as well.

### PPI reduced PTX resistance in EOC cells through the inhibition of YAP. Synergetic effects of the combination of PPI and PTX resulted in the inhibition of YAP and cytotoxicity to PTX-resistant EOC cells

**Fig. 4:**
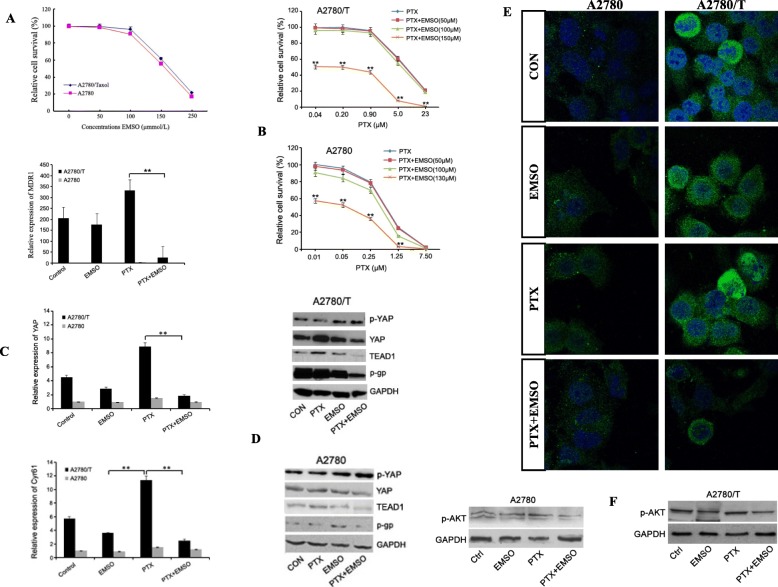
PPI reduced PTX resistance in EOC cells through the inhibition of YAP. Synergetic effects of the combination of PPI and PTX resulted in the inhibition of YAP and cytotoxicity to PTX-resistant EOC cells. Two EOC cells (A2780 and A2780/T) were treated with different concentrations of either PTX or combinated with sub-lethal doses of EMSO (130 and 150 μM) for 48 h. **a** Dose-response curve of EMSO treatment in the two EOC cell lines. **b** Cell viability is shown after PTX treatments with or without EMSO for 48 h using CCK-8 assay in PTX-sensitive A2780 cells and PTX-resistant A2780/T cells. Each value represents the mean ± SD from three independent experiments. **c** The mRNA level of MDR1, Cyr61 and YAP were determined using a real-time PCR kit. Data represent mean ± SD from three independent experiments (**P < 0.01). **d** The expressions of p-gp, TEAD1, YAP and p-YAP protein were detected by Western blot analysis. **e** YAP expression on the cytoplasm and nuclear of cells is shown using green staining. Cell immunofluorescence was detected by confocal microscopy. Magnification × 600; Nuclear staining with DAPI is shown in blue. Representative images from three independent experiments are shown. **f** The expressions of p-AKT was detected by Western blot

The cytotoxic effect of EMSO was investigated by employing a CCK-8 assay on A2780/T and A2780 cells. We found that EMSO treatment of A2780/T and A2780 cells resulted in anti-growth effects at sub-lethal doses in both cell types. The sub-lethal doses of EMSO were approximately 150 and 130 μM in the A2780/T and A2780 cells, respectively (Fig. 4a). The decrease in the percentage of viability was statistically significant at low doses of PTX when combined with sub-lethal doses of EMSO (*P* < 0.01). Even at the lowest dose of PTX, 0.01 and 0.04 μM, the combination with sub-lethal doses of EMSO (150 and 130 μM) decreased viability dramatically, whereas PTX alone or in combinations with 50 μM EMSO were ineffective in two cell types (Fig. 4b). The growth inhibitory effects of 130 μM EMSO+PTX were significant at 0.01, 0.05, 0.25 and 1.25 μM in A2780 cells. On the other hand, the growth inhibitory effects of 150 μM EMSO+PTX were significant at 0.04, 0.20, 0.90, 5.0 and 23 μM in A2780/T cells. However, PTX combinations with 100 μM EMSO did not result in a higher efficacy (Fig. 4b). We further examined if there were any synergistic, additive, or antagonistic interactions between EMSO and PTX. As expected, the resulting interaction of sub-lethal doses of EMSO combinated with PTX was synergism at all doses except for the 130 μM EMSO+ 7.50 μM PTX combination in A2780 cells, which was an additive interaction (Table 3).

**Table 3:** Paclitaxel and EMSO Combination Index (CI) against A2780 and A2780/T Cells

Combination type	Inhibitory rate	CI	Interaction type
A2780+ PTX(0.05)	4.7		
A2780+ EMSO(50)	1.6		
A2780+ PTX(0.05) + EMSO(50)	6.3	1.0	Additive
A2780+ PTX(0.25)	29.1		
A2780+ EMSO(100)	9.2		
A2780+ PTX(0.25) + EMSO(100)	38.3	1.0	Additive
A2780+ PTX(1.25)	72.3		
A2780+ EMSO(130)	44.2		
A2780+ PTX(1.25) + EMSO(130)	95.3	0.94	Synergistic
A2780/T+ PTX(0.04)	0.7		
A2780/T+ EMSO(50)	1.2		
A2780/T+ PTX(0.04) + EMSO(50)	2.0	1.0	Additive
A2780/T + PTX(0.90)	1.6		
A2780/T+ EMSO(100)	2.7		
A2780/T + PTX(0.90) + EMSO(100)	4.3	1.0	Additive
A2780/T + PTX(5.0)	24.3		
A2780/T+ EMSO(150)	43.9		
A2780/T + PTX(5.0) + EMSO(150)	85.4	0.91	Synergistic

CI < 1, synergistic; CI =1, additive; or antagonistic,CI > 1

We also demonstrated the specific and significant inhibition of PTX-resistance related mRNA and protein expression in A2780 and A2780/T exposed to PTX (0.25 and 2 μM), EMSO (130 and 150 μM) alone or to the combination with PTX. MDR1 expression was very high in the A2780/T cells, but nearly absent in the parental A2780 cells. The levels YAP and cyr61 expression in the A2780/T cells co-treated with EMSO+PTX were significantly decreased, compared to EMSO and PTX alone treatment. However, statistical significance was not achieved neither for YAP nor cyr61 expression in A2780 cells using the same treatment combination (Fig. 4c). As shown in Fig. 4d-e, compared to YAP expression, p-gp expression was very high in the A2780/T cells, but nearly absent in the parental A2780 cells. Q-PCR and confocal analysis revealed that YAP and its downstream target Cyr61 was inhibited by combination therapy with sub-lethal doses of EMSO and PTX in the A2780/T cells, compared to EMSO and PTX alone (Fig. 4d-e). Moreover, the increase of p-YAP expression and decrease of p-gp and TEAD1 expression in the two ovarian cancer cells were reversed by a co-treatment with EMSO+PTX (Fig. 4d).

It has been well documented that the PI3K/AKT pathway is essential for the proliferation and invasion of tumor cells, and activation of the PI3K/AKT signaling pathway is regulated by YAP. Western blots of two cell lysates showed that a combination treatment with PTX + EMSO did affect the phosphorylation status of Akt (Fig. 4f). Therefore, co-treatment with PTX + EMSO may decrease YAP nuclear localization and activity and causes more sensitivity to PTX in the two ovarian cancer cells.

### PPI-mediated apoptosis was revealed using a caspase-independent apoptotic-like cytotoxicity upon PTX treatment of EOC cells

**Fig. 5:**
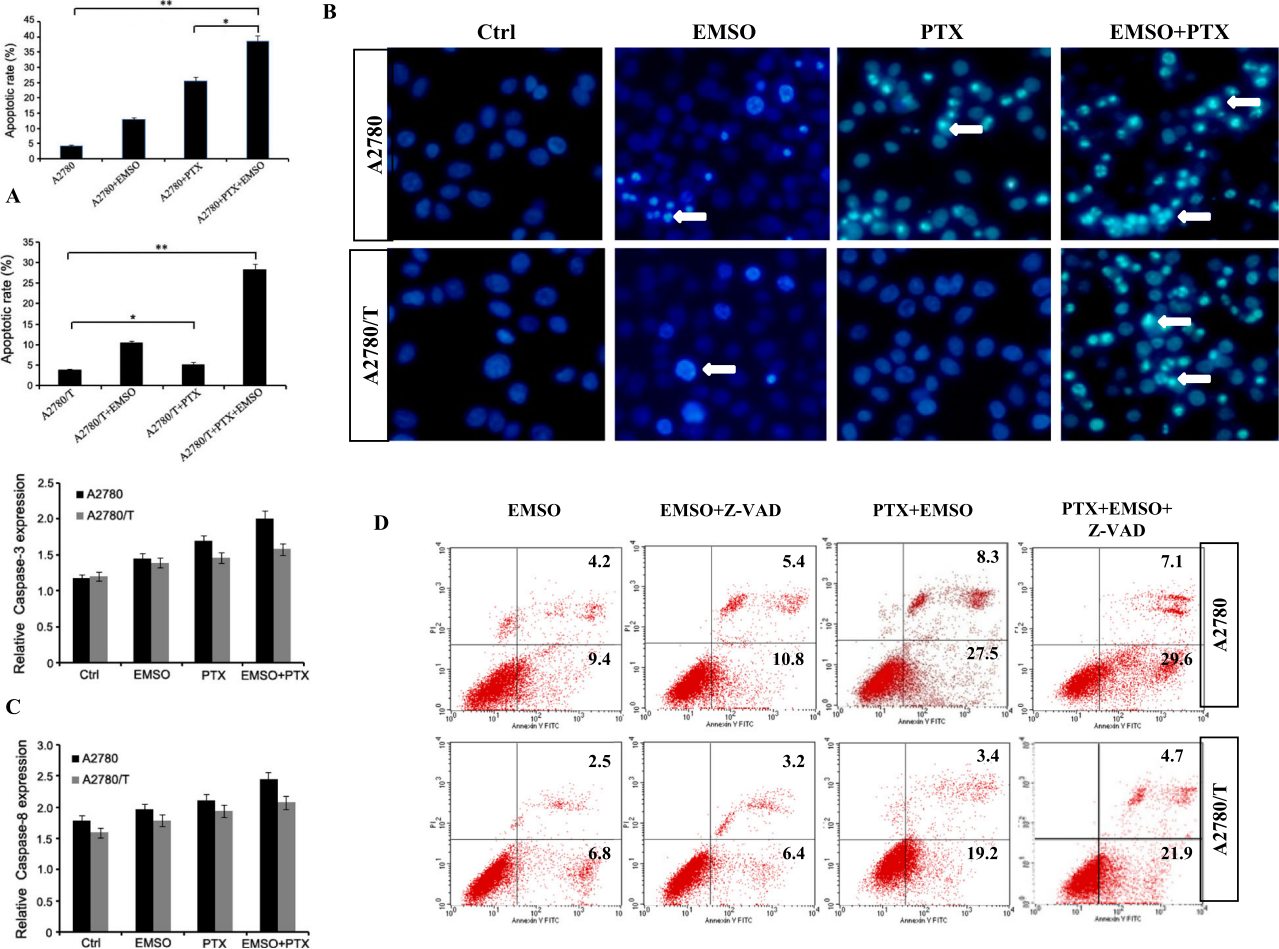
PPI-mediated apoptosis was revealed using a caspase-independent apoptotic-like cytotoxicity upon PTX treatment of EOC cells. **a** Quantification of apoptotic cells: The apoptosis ratio is the ratio of the number of cells in early-stage apoptosis to that in advanced stage apoptosis. Data are presented as the mean ± SD from three independent experiments (*P < 0.05, **P < 0.01). **b** Images of fluorescent cells were taken after staining with Hoechst-33342 following treatment with EMSO (130 or 150 μM), PTX (0.25 or 2 μM), PTX (0.25 or 2 μM) + EMSO (130 or 150 μM) for 48 h. The white arrows denote early-stage apoptotic nuclei (× 400). **c** Expression of active caspase-3 and caspase-8, as measured by an ELISA assay. Data (mean ± SD) are from three independent experiments. **d** Cytograms obtained for A2780 and A2780/T cells following a 48 h- continuous exposure to EMSO (130 or 150 μM), or PTX (0.25 or 2 μM) + EMSO (130 or 150 μM), in the absence and presence of Z-VAD-fmk

To assess cell apoptosis in A2780 and A2780/T cells, apoptosis rate was measured by flow cytometry and Hoechst-33342 with PTX (0.25 and 2 μM) alone and in combination with EMSO (130 and 150 μM). We found that the apoptosis rate in the two EOC cells induced by sub-lethal doses of EMSO was about 2-fold than that of untreated cells, suggesting that the specific cytotoxic effects of EMSO in EOC cells were apoptosis-related. EMSO co-treatment show synergistic effect on PTX-induced apoptosis in two ovarian cancer cells. Interestingly, the increase rate in apoptosis with EMSO was higher in PTX-resistant A2780/T cells than in PTX-sensitive A2780 cells (Fig. 5a-b). Afterwards, A2780/T cells restored PTX sensitivity.

We next examined the possible mechanisms responsible for the apoptotic effects of sub-lethal doses of EMSO combined with PTX, we evaluated its effects based on the activation of caspases. Morphological analysis showed that sub-lethal doses of EMSO combined with PTX had no significant effect on caspase-3 and caspase-8 activities in the two EOC cell lines (Fig. 5c). To confirm the role of caspases in sub-lethal doses of EMSO combination with PTX-induced cell death, the pan-caspases inhibitor z-VAD-fmk was used at 20 μM (Fig. 5d). We observed that preincubation with z-VAD-fmk, had no significant effect on co-treatment PTX + EMSO-induced apoptosis in the two EOC cells. This suggests that activated caspases are not instrumental for the combination of EMSO with PTX-induced apoptosis in the EOC cells.

### Autophagy flux inhibited by the combinations of PTX with sub-lethal doses of EMSO

**Fig. 6:**
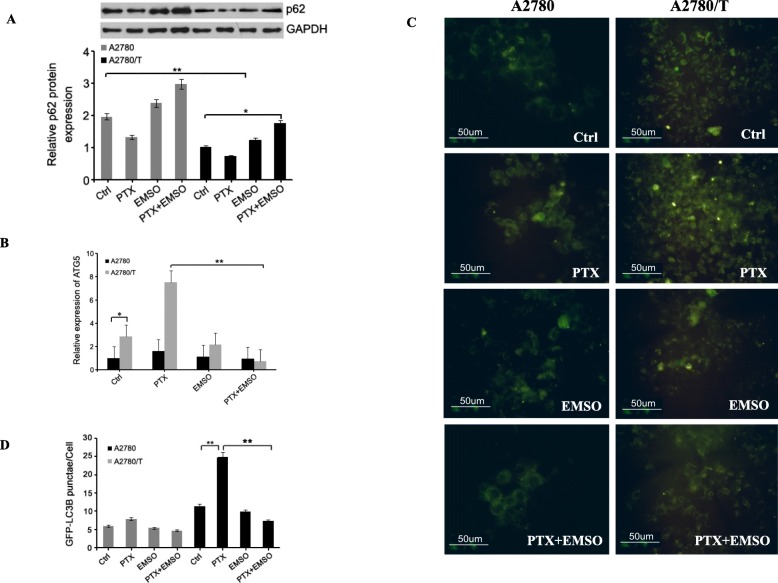
Autophagy flux inhibited by the combinations of PTX with sub-lethal doses of EMSO. **a** Representative Western blot showing the relative levels of p62 protein (*N* = 3). Densitometric analysis of the levels of p62 protein is shown in the bottom in A. Gray column stands for A2780 cells, black column represents A2780/T cells (*P < 0.05). **b** The mRNA level of ATG5 was determined using a real-time PCR kit. Data represent the mean ± SD from three independent experiments (*P < 0.05). **c** Autophagy evaluated by GFP-LC3B puncta formation in two EOC cells transfected with an Ad- mCherry-GFP-LC3B construct for 48 h. **d** Quantification of GFP-LC3B puncta showing means + SE using Image Pro software (Version premier). A representative experiment is shown and quantification is based on three repeats (**p < 0.01 vs control). IC50 values of paclitaxel, EMSO plus paclitaxel, and EMSO as well as combination index values of synergistic activity in A2780 and A2780/T cell lines

In addition, we determined if the level of autophagy flux was inhibited by the combination of PTX and sub-lethal doses of EMSO with the autophagy marker p62 used as an indicator of autophagic flux whose levels would decrease when there was an active autophagic flux within the cell [15]. To investigate the role of PTX in inducing autophagy, we evaluated the levels of p62 protein in the A2780 and A2780/T cells exposed to PTX (0.25 and 2 μM), EMSO (130 and 150 μM) alone or in combination after 48 h. Our results showed that PTX-resistant A2780/T cells expressed significantly lower levels of p62 when compared with the PTX-sensitive A2780 cells. Importantly, the levels of autophagy-related proteins 5 (ATG5) were higher in A2780/T when compared with A2780 cells. Inversely, co-treatment with sub-lethal dose of EMSO for 48 h induced a modest increase in p62 protein expression in the A2780/T cells (Fig. 6a). We further demonstrated that suppression of PTX induced autophagy by sub-lethal doses of EMSO combined with PTX in the A2780/T cells, was associated with a noticeable decrease in the ATG5 (Fig. 6b). But the same effect was not confirmed in the A2780 cells. Thus, co-treatment with sub-lethal doses of EMSO inhibited autophagy and enhanced PTX cytotoxicity. Actually, overexpression of ATG5 not only promotes autophagy but also promotes apoptosis [16]. Further work is required to understand how ATG5 in regulating pro-apoptotic signaling pathways.

We also found that PTX treatment preferentially induced GFP-LC3B puncta in the A2780/T cells but not in the A2780 cells. There was also an increased accumulation of LC3B in the presence of a sub-lethal dose of EMSO combined with PTX with a strongly enhanced the presence of punctuate fluorescence in A2780/T cells (Fig. 6c-d), indicating ESOM partially reduced autophagic flux.

These findings suggest that co-treatment with a sub-lethal dose of EMSO likely causes the accumulation of autophagosomes and slows down the autophagic flux.

## Discussion

Acidic microenvironment increased by overexpression of V-ATPases has been observed in malignant tissues. It was also found that higher expression of V-ATPase mRNA was associated with a significant poor survival [17]. Our results revealed that V-ATPase D1 silencing cells exhibited 2.25 -fold inhibition in YAP protein level. The inhibition of V-ATPase D1 reduces YAP activities in A2780/T cells. Knocking down the expression of V-ATPase D1 in A2780/T cells prevented the expression of target proteins downstream of YAP. Overall, our studies have provided a novel mechanism in PTX-resistance ovarian cancer cells through the activating of YAP transcriptional activity by V-ATPase D1.

Previous research has reported that over-expression of YAP promotes PTX-resistance in ovarian cancer cells [18]. The precise mechanisms by which YAP enhances drug resistance remain unclear. In the present study, we found that YAP was significantly elevated in A2780/T cells, compared with A2780 cells. To further confirm the relationship between YAP and PTX resistance, over-expression of YAP by stable transfection enhanced the expression level of YAP and contributed to PTX -resistance in the A2780 cells. Correspondingly, suppression of YAP by siRNA effectively inhibited YAP expression, resulting in PTX-sensitivity in A2780/T cells. Our immunostaining results also showed that TEAD1 was positively correlated with YAP expression, suggested that TEAD1was a direct binding partner of YAP. Therefore, we speculate that modulating YAP and TEAD1 activities may be an effective strategy to prevent the PTX-resistance in ovarian cancers.

Treatment using PPIs has been proposed as a valid and feasible approach because of the relatively low toxicity and potential selectivity of these drugs. It has been reported that PPIs are chemosensitizing as well as cytotoxic drugs, active against several human tumor cells, such as osteosarcoma [19, 20], melanoma [21, 22], gastric carcinoma [23–25], pancreatic cancer [26] and breast cancer [27–29]. In this context, we hypothesized that the V-ATPase blocker PPI could reverse the YAP-mediated PTX-resistance in EOC cells. Our results demonstrated that a sub-lethal dose of EMSO affected the membrane V-ATPase D1 expression, as well as regulated the transmembrane pH gradient in EOC cells, suggesting that V-ATPase D1 mediated the acidic tumor microenvironment formation. These data are in agreement with one study demonstrating in MCF-7 breast carcinoma cell line that a low extracellular pH induces higher resistance to PTX [30].

When used in combination, the PPIs improve the efficacy of the anti-cancer drugs. This study further supports these previous observations. In a lung cancer study, it has been proven that the combination of EMSO with chemotherapeutics results in a more pronounced cytotoxic effect [31]. In our study, apparent and statistically significant cytotoxic effects were observed in a combinated treatment with a sub-lethal dose of EMSO and PTX. We also found that PTX combined with sub-lethal doses of EMSO generated strong synergism effect. A sub-lethal dose of EMSO could effectively inhibit YAP protein and its down-stream proteins. Moreover, our results showed that the down-regulation of MDR1/P-gp protein expression. In addition, we observed that the decrease in phosphor-AKT levels was accompanied by lower cell survival rates and PTX-sensitivity in A2780 and A2780/T cells exposed to a PTX + EMSO co- treatment. Thus, our results indicated a substantial reversal in PTX-resistance cells, was YAP-mediated in EOC cells.

To improve treatment efficacy, it is crucial to investigate the precise molecular mechanism of PPIs in EOC, which may occur through: (i) inhibition of drug extrusion from cells via P-glycoprotein or other MDR-related mechanisms [18]; (ii) increase in anti-apoptotic molecules [32]; and (iii) acidic endosomes, which are an important step in the maturation of autophagosomes and PPIs which inhibit autophagy [33]. Some human cancers, including ovarian, gastrointestinal, prostate and breast cancers [34–37] exhibit high levels of autophagic activity. Studies have described the induction of autophagy in response to therapeutic agents [38]. For example, cisplatin and PTX were able to induce autophagy in A549 lung cancer cells [39]. Increasing evidence suggests that inhibition of autophagy augments cytotoxicity caused by anticancer drugs in preclinical models [40]. Inhibitors of autophagy that can be used in vivo include CQ (chloroquine), HCQ (hydroxychloroquine) and PPIs. Co-treatment with pantoprazole was able to inhibit autophagy and enhance cytotoxicity in vivo [41].

On the search for cellular mechanisms that mediate the effect of EMSO in EOC cell PTX-resistance, our data are consistent with other recent studies indicating that resistance to PTX is associated with up-regulation of autophagy in EOC cells. The administration of EMSO can augment the activity of PTX, but only at sub-lethal doses. EMSO combination of PTX has been shown to be feasible with a good toxicity profile. An increase in cell death was observed when PTX was combinated with sub-lethal doses of EMSO. We also showed that sub-lethal doses of EMSO exerts their cytotoxic action through a caspase-independent apoptotic-like cell death.

Despite our important findings, our study has several limitations. This analysis was limited to two selected EOC cells and one basic anticancer drug. We also did not perform an in vivo study to assess the potential clinical relevance of the in vitro results.

## Conclusions

Clinical and experimental approaches were combined to demonstrate that V-ATPase D1 regulated YAP expression mediated PTX-resistance in EOC. To this end, drugs targeting the V-ATPase D1/YAP signaling pathway can be specifically developed to overcome the occurrence of tumor resistance in EOC. Thus, EMSO may be useful as a chemosensitizer in the treatment of patients with PTX-resistance EOC.

## Methods

### Patients and tissue specimens

Tumor samples from patients with ovarian cancer were acquired from the First Affiliated Hospital of An Hui Medical University (PJ2017-0704) and a written informed consent was approved by the First Affiliated Hospital of An Hui Medical University Institutional Review Board. Twelve normal ovarian specimens were obtained during surgery from women with myoma of uterus. A single gynecologic pathologist (I-G Do) examined the specimens using hematoxylin and eosin staining. Specimens were selected in analysis if they comprised more than 90% tumor cells. Data was collected from electronic and paper medical records.

### Reagents

Esomeprazole sodium was purchased from AstraZeneca (Sweden) and dissolved to 10 mmol/L in saline before use. PTX injection was purchased from Aosaikang Co., Ltd. (Jiangsu, China). Z-VAD-FMK was purchased from Calbiochem (USA).

### Cell lines and culture

Human EOC cell lines (A2780 and A2780/Taxol) were purchased from Hui Ying Biological Technology Co., Ltd., China. PTX-sensitive A2780 Cells was maintained in complete DMEM, high glucose (Hyclone, USA) supplemented with 10% fetal bovine serum. PTX-resistant A2780/Taxol (A2780/T) cells were cultured in RPMI 1640 supplemented with 10% fetal bovine-serum medium, first the A2780/T cells were exposed to 100 nM PTX for 24 h, then the medium was changed to fresh one without PTX. When cells reached almost 80% confluency, the cells were incubated with increasing concentrations of PTX to finally obtain the cells resistant to 1200 nM PTX. For maintenance of PTX-resistance phenotype, 250 nM PTX was added into the normal medium. The drug resistant cells were cultured in PTX-free medium for at least 2 weeks prior to being used in experiments. All cells were cultured at 37 °C in a humidified atmosphere incubator with 5% CO2.

### Immunohistochemical analysis

Immunohistochemical studies were carried out on formalin-fixed, paraffin- embedded, 4 μm thick tissue sections and stained with hemoxylin and eosin. After deparaffinization and antigens retrieval, slides were washes, blocked and incubated with V-ATPase D1(Santa Cruz, USA) and YAP (Santa Cruz, USA) at 4 °C overnight, followed by 1 h incubation with biotinylated goat-anti-mouse or biotinylated goat-anti-rabbit secondary antibody. Immunostaining was performed with diaminobenzidine (DAB) using the streptavidin-biotin complex/horseradish peroxidase (sABC-HRP) method. Sections were counterstained with hematoxylin, and then mounted in medium. For each sample four microscopic fields (× 200) in three different sections were counted. The intensity of staining was graded on a semi quantitative scale from 0 to 3, where 0 = no staining, 1+ = weak staining, 2+ = moderate staining, and 3+ = strong staining. The range of possible scores was between 0 and 250.

### Cell transfection

To figure out if YAP directly regulated by V-ATPase expression, we generated stable V-ATPase D1 knockdown YAP-high/PTX-resistant A2780/T cells by transfection of V-ATPase D1 shRNA plasmid (Santa Cruz, USA). Stable clones were selected with G418 (Gibco, USA). To investigate the relationship between YAP and PTX resistance, YAP plasmids and siYAP-RNA were transfected, respectively, into YAP-low/PTX-sensitive A2780 cells or YAP-high/PTX-resistant A2780/T cells, using Lipofectamine 2000 transfection reagent (Invitrogen, USA) as per manufacturer’s instructions. After incubation for 48 h, the transfected cells were used for the following experiments.

### Luciferase assay

Human YAP cDNA was inserted into the expression vector pCL3-based verctos, resulting in the Flag-tagged YAP (Flag-YAP). YAP wild type or S94A mutant, and TEAD1 shRNAs were designed in a region identical in TEAD1, then stably co-transfected with a Renila luciferase expression plasmid into cells. Luciferase activities were analyzed using a dual-luciferase reporter kit (Promega, USA). Transfections were performed in triplicate and repeated three times to ensure reproducibility.

### Measurement of intracellular pH levels

BCECF-AM (Beyotime Biotechnology, China) is the most widely used fluorescent indicator for intracellular pH. The BCECF-AM was excited at 488 nm with emission collected at 535 nm. Cells (1 × 10^5^ cells) were seeded in a 35-mm confocal dish in RPMI1640 and high glucose DMEM with 10% FBS. The next day, cells were exposed to EMSO in serum free RPMI 1640 and high glucose DMEM. After 48 h, the cells were treated with 1 μmol/L BCECF-AM solution, incubated at 37 °C for 40 min and analyzed by fluorescence microscopy.

### Immunofluorescence assay

Cells were grown on round glass coverslips (Thermo Fisher Scientific, USA) in 35 mm cell culture dishes. Following a 7-9 min fixation with 4% pre-chilled paraformaldehyde, the coverslips were washed with PBS, permeabilized with 0.5% Triton X-100-PBS for 5-10 min, and blocked with 5% bovine serum albumin-PBS for 60 min. The YAP antibody was incubated with the cells overnight at 4 °C. After incubation with the appropriate secondary fluorescent antibody Alexa green 488 nm (Molecular Probes, Life Technologies, USA), nuclei were stained with DAPI (Beyotime Biotechnology, China) and the cell were immediately observed under a fluorescence microscope. For the co-localization analysis of V-ATPase D1 with plasma membrane, cells were washed and fixed as described above, but not permeabilized. After the immunofluorescence stain for the V-ATPase D1, cells were also stained with a pan-cadherin (Abcam, UK) that bind of the cell membrane.

### Cytotoxicity of a single and combined drug in PTX sensitive and resistant EOC cells

Cell cytotoxicity was analyzed with Cell Counting Kit-8 (CCK-8) assay kit (Beyotime Biotechnology, China). Briefly, cells were seeded into flat-bottomed 96-well plates at a density of 8000 per well and incubated overnight. After cellular adhesion, the A2780/T cells were exposed to various doses of PTX (0.04, 0.2, 0.9, 5, 23 μM) and in the absence or presence of EMSO (150 μM), the A2780 cells were exposed to various doses of PTX (0.01, 0.05, 0.25, 1.25, 7.5 μM) in the absence or presence of EMSO (130 μM) for 48 h followed by treatment with 10 μl of CCK-8 solution for an additional 1 h at 37 °C, and the absorbance was measured at 450 nm using a microplate reader (Perkin Elmer, USA). At least three independent experiments were performed.

### Cell apoptosis detection

Apoptosis was induced by PTX (0.25 and 2 μM) with or without EMSO (130 and 150 μM) co-treatment for 48 h each. Apoptosis detection was carried out with Annexin V-FITC and Propidium Iodide (PI) double staining assay kit (KeyGen Biotech, China), following the manufacturer’s instruction. Apoptotic cells were uncovered using flow cytometry (BD Bioscience, USA). Data acquisition and analysis were done with CellQuest V3.3 (BD Bioscience, USA).

### Morphological detection of apoptosis by Hoechst-33342 assay

Cells were harvested and washed in ice-cold PBS and fixed with 3.7% paraformaldehyde in PBS for 10 min at room temperature. Fixed cells were permeabilized with saponin and stained with Hoechst-33342 solution (Beyotime Biotechnology, China) for 10 min at room temperature. Nuclear morphology of cells was examined by fluorescence microscopy. The percentage of apoptosis was calculated by counting the condensed and fragmented nuclei in cells. The experiment was repeated three times.

### Green fluorescent protein-LC3 translocation

Ad-mCherry-GFP-LC3B (Beyotime Biotechnology, China) was used to detect autophagy. Cells were transfected with Ad-mCherry-GFP-LC3B using Lipofectamine 2000 in serum-free medium for 240 min after which the medium was changed to a complete medium. After 24 h, the transfected cells were exposed to PTX (0.25 and 2 μM) in the absence or presence of EMSO (130 and 150 μM) for 48 h. The distribution and the fluorescence of mCherry-GFP-LC3B were visualized by fluorescence microscopy.

### Real-time reverse transcription PCR

Total RNA was extracted with Trizol Reagent (Invitrogen, USA) and quantified by a spectrophotometer. cDNA synthesis was carried out using M-MLV reverse transcriptase (Invitrogen, USA). Quantitative PCR experiments were performed on the 7500 Real-time PCR system (Applied Biosystems, USA) by using the SYBR Green PCR reagents (Takara, Japan). The levels of mRNA were calculated using the equation 2-∆∆CT and normalized to human 18 s mRNA levels.

### Western blotting analysis

The total protein was extracted using RIPA lysis buffer (Beyotime Biotechnology, China) supplemented with protease inhibitors cocktail (Roche, USA). The proteins were quantified using BCATM Protein Assay Kit (Pierce, Appleton, USA). The western blot was performed according to the standard protocol.

### Enzyme-linked immunosorbent assay

For the analysis of active caspase-3 and caspase-8 expression, cells were lysed in PRO-PRE Protein Extraction Solution (Intron Biotechnology, Korea). The protein concentration was determined using a BCA protein kit (Thermo Scientific, USA). The concentrations of active caspase-3 and caspase-8 (Invitrogen, USA) were determined using an Enzyme-linked immunosorbent assay (ELISA) kits used according to the manufacturer’s instructions. All samples were measured in triplicate.

### Statistical analysis

The correlation between YAP and V-ATPase D1 was evaluated using Spearman’s rank correlation coefficient test. The results are expressed as the mean + standard error (SE) or standard deviation (SD) of the raw data. The Student’s t test was used to determine the statistical significance of differences between the control and test groups. A *p* value of < 0.05 was considered statistically significant.

Combination index (CI) values were calculated using CalcuSyn Version 2.1 according to the Chou-Talalay method for drug combinations (Chou, 2010). CI is a parameter that gives information about the effectiveness of drug combinations. Combination effects are defined as very strong synergism (CI < 0.1), strong synergism (0.1 < CI < 0.3), synergism (0.3 < CI < 0.7), moderate synergism (0.70 < CI < 0.85), slight synergism (0.85 < CI < 0.90), nearly additive (0.9 < CI < 1.1), or antagonistic (CI > 1.1) (Chou, 2006).

## Data Availability

Not applicable.

## Abbreviations

ATG5: Autophagy-related proteins 5
CCK-8: Cell Counting Kit-8
CI: Combination index
ELISA: Enzyme-linked immunosorbent assay
EMSO: Esomeprazole
IC50: Half maximal inhibitory concentration
Paclitaxel: PTX
PI: Propidium Iodide
PPI: Proton pump inhibitors
SE: Standard error
ShRNA: Short hairpin RNA
Sh-scr: Scrambled short hairpin RNA
